# Baloxavir Acid-Induced Mitochondrial Toxicity and Cell Cycle Arrest Contribute to Its Adverse Effects

**DOI:** 10.3390/ijms27072967

**Published:** 2026-03-25

**Authors:** Pengyu Zhan, Yuxing Ren, Kai Han, Guoming Jin, Yang Yang, Lei Shi, Yali Ci

**Affiliations:** 1 State Key Laboratory of Common Mechanism Research for Major Diseases, Institute of Basic Medical Sciences & School of Basic Medicine, Chinese Academy of Medical Sciences & Peking Union Medical College, Beijing 100005, China; zhanpybjfu@163.com (P.Z.); 15035889732@163.com (Y.R.); hankai8435@163.com (K.H.); 13037523540@163.com (G.J.); yangy@ibms.pumc.edu.cn (Y.Y.); 2 Department of Biochemistry and Molecular Biology, Institute of Basic Medical Sciences & School of Basic Medicine, Chinese Academy of Medical Sciences & Peking Union Medical College, Beijing 100005, China

**Keywords:** baloxavir, mitochondria, cytotoxicity

## Abstract

Baloxavir has emerged as a breakthrough anti-influenza therapy, owing to its single-dose regimen and rapid viral clearance. Nevertheless, clinical adverse effects have been reported, while the underlying cellular mechanisms remain unclear. In this study, we demonstrate that baloxavir acid rapidly induces mitochondrial morphological abnormalities. This mitochondrial dysfunction subsequently initiates a cascade of cellular events, including G0/G1 cell cycle arrest mediated by the downregulation of cyclin D3 and CDK4, and apoptosis via the Bak-caspase-3 pathway. Co-treatment with the antioxidant N-acetylcysteine alleviated baloxavir-induced mitochondrial abnormalities and the decreased expression level of cyclin D3. In contrast, the prodrug baloxavir marboxil exhibited minimal mitochondrial toxicity, underscoring the advantage of the prodrug strategy in reducing adverse effects. Our findings identify mitochondrial impairment as a key mechanism for baloxavir-induced cytotoxicity and provide molecular insights that may help explain its clinical adverse profile.

## 1. Introduction

Influenza remains a major global health challenge, affecting all regions and causing significant morbidity and mortality [1,2,3]. According to the World Health Organization (WHO), approximately one billion people are infected annually, resulting in 3–5 million severe cases and an estimated 290,000–650,000 respiratory-related deaths [4]. The continuous antigenic evolution of influenza viruses enables them to evade immunity acquired from prior infection or vaccination, highlighting the need for ongoing therapeutic development [5]. Beyond vaccination, antiviral drugs are critical for influenza management [6]. Current antivirals against influenza include M2 ion channel inhibitors, RNA-dependent RNA polymerase (RdRp) inhibitors, neuraminidase inhibitors, and endonuclease inhibitors [7,8,9].

Among these, baloxavir (marketed as Xofluza), approved by the FDA in 2018, represents a breakthrough as a cap-dependent endonuclease inhibitor that has reshaped treatment paradigms and gained substantial market presence [10,11,12]. It suppresses viral replication by specifically inhibiting the endonuclease activity of the viral polymerase acidic (PA) protein, thereby blocking the “cap-snatching” process essential for viral mRNA synthesis [13,14]. This mechanism targets the initial stage of viral transcription, complementing neuraminidase inhibitors and offering a new strategic option for influenza control [15,16,17,18,19]. Clinical advantages of baloxavir include a single-dose regimen, rapid viral clearance, and a novel mechanism that may synergize with existing antivirals [20]. Baloxavir marboxil (BXM), a prodrug designed to enhance oral bioavailability, is rapidly hydrolyzed by esterases in the intestine and liver to form the active metabolite baloxavir acid (BXA) [21].

With increasing clinical use, however, some adverse effects of baloxavir have been reported (occurring in approximately 5% of patients) [12], including diarrhea, bronchitis, nausea, headache [22,23], hemorrhagic complications [24,25], hepatic dysfunction, rhabdomyolysis [26,27] and cardiorespiratory events [28]. Pediatric patients and those on anticoagulant therapy may require closer monitoring due to higher risks of bleeding, neurological effects (including febrile delirium), and gastrointestinal disturbance [28,29,30,31,32]. Despite recognition of these adverse effects, the cellular response and underlying molecular mechanisms remain poorly understood.

Here, we demonstrate that baloxavir acid induces mitochondrial abnormalities and subsequent cell cycle perturbation. Using quantitative morphological analysis and live-cell time-lapse imaging, we show that baloxavir acid triggers rapid and pronounced mitochondrial swelling and fragmentation. These damaged mitochondria subsequently lead to cell cycle arrest and apoptosis, mediated through the cyclin D3/CDK4 downregulation and activation of the Bak-caspase-3 pathway. In contrast, the prodrug baloxavir marboxil exhibited minimal cytotoxicity, underscoring the value of the prodrug strategy in mitigating adverse effects. Our findings elucidate baloxavir acid-induced cytotoxicity and its underlying mechanism, which may help explain its clinical adverse effects.

## 2. Results

### 2.1. Baloxavir Acid (BXA) Induces Mitochondrial Fragmentation and Dysfunction

**Figure 1:**
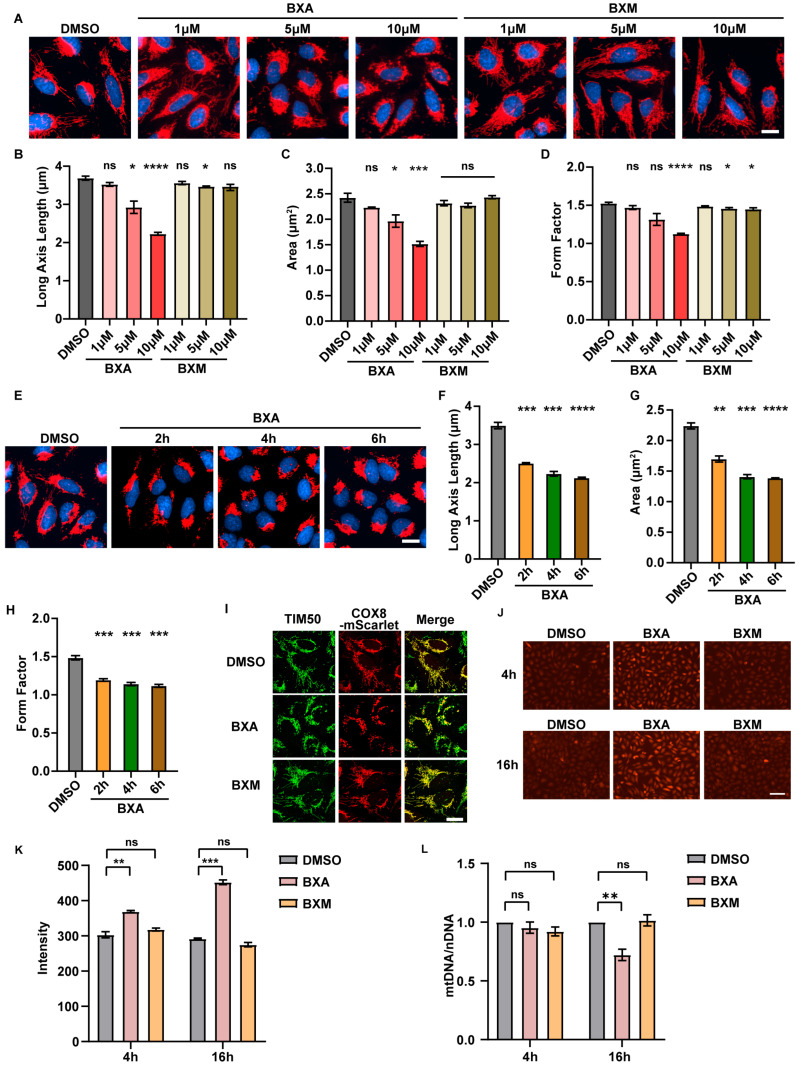
BXA induces pronounced mitochondrial morphological abnormalities and leads to mitochondria functional damage. (**A**) Representative images showing mitochondrial morphology in U2OS cells treated with BXA or BXM. Mitochondria were stained with TMRM (red) and nuclei with Hoechst (blue). (**B**–**D**) Quantitative analysis of mitochondrial morphology parameters corresponding to (**A**), including long-axis length (**B**), area (**C**), and form factor (**D**), performed using CellProfiler software 4.2.6. (**E**) Representative images showing mitochondrial morphology upon 10 μM BXA treatment over the time (U2OS cells). Mitochondria were labeled with TMRM (red) and nuclei with Hoechst (blue). (**F**–**H**) Quantitative analysis of mitochondrial morphology parameters corresponding to (**E**), including long-axis length (**F**), area (**G**), and form factor (**H**). (**I**) Mitochondrial morphology in U2OS cells treated with BXA or BXM, visualized using the fluorescent mitochondrial marker cytochrome c oxidase subunit 8-mScarlet (COX8-mScarlet) and endogenous mitochondrial marker TIM50. (**J**) Representative images of Rhod-2 AM staining after 4 h or 16 h of drug treatments. (**K**) Quantification of Rhod-2 AM fluorescent intensity from (**J**). (**L**) Ratio of mitochondrial DNA to nuclear DNA (mtDNA/nDNA) following drug treatments. Scale bars: 20 μm (**A**,**E**,**I**), 200 μm (**J**). Data are presented as mean ± SEM from three independent experiments. ns, not significant; * *p* < 0.05, ** *p* < 0.01, *** *p* < 0.001, and **** *p* < 0.0001 vs. control group.

In a previous high-content live-cell screen assessing mitochondrial morphology in U2OS cells after drug treatment, we observed that BXA caused pronounced mitochondrial fragmentation. To further investigate, we conducted a comprehensive quantitative analysis of mitochondrial morphology using escalating concentrations (1, 5 and 10 μM) of BXA and its prodrug, baloxavir marboxil (BXM), for 4 h. Live-cell imaging was performed using the cell-permeant mitochondrial dye TMRM to visualize mitochondrial morphology (Figure 1A). Results showed that BXA triggered mitochondrial fragmentation in a dose-dependent manner. Automated image analysis of multiple morphometric parameters revealed significant dose-dependent reductions in mitochondrial length, area, and form factor (inverse of circularity) (Figure 1B–D). In contrast, the prodrug BXM showed no apparent effects even at 10 μM. Similar results were observed in HeLa cells treated for 24 h (Supplementary Figure S1A). The marked difference in mitochondrial effects between BXA and its prodrug underscores the importance of drug bioactivation in mediating mitochondrial toxicity and highlights a key advantage of the prodrug formulation in reducing toxicity, a finding not previously reported. Temporal kinetics analysis indicated that BXA-induced mitochondrial fragmentation began as early as 2 h post-treatment and progressed over time (Figure 1E–H), indicating rapid and potent cellular effects. To validate these results and exclude potential artifacts from TMRM staining, we examined mitochondrial morphology using COX8-mScarlet (a mitochondrial-targeted fluorescent protein) [33] and TIM50 immunostaining (an endogenous mitochondrial marker) [34]. Both approaches confirmed that BXA, but not BXM, induced mitochondrial fragmentation (Figure 1I).

Since heterogeneous mitochondrial morphologies are often associated with mitochondrial dysfunction, we further examined calcium levels, membrane potential, and mtDNA content following BXA treatment. To monitor changes in mitochondrial calcium handling, we utilized the mitochondria-targeted calcium-sensitive fluorescent dye Rhod-2 AM. Rhod-2 AM fluorescence intensity was elevated as early as 4 h post-treatment, and by 16 h had risen to approximately 1.5-fold of that in control cells, indicating progressive disruption of mitochondrial calcium homeostasis (Figure 1J,K). Regarding mitochondrial membrane potential, although 4 h of BXA exposure induced aberrant mitochondrial morphologies including fragmentation, no significant change in the JC-1 red/green fluorescence ratio was observed compared to controls (Supplementary Figure S1B,C). In contrast, 16 h of BXA treatment resulted in an approximately 50% reduction in the JC-1 red/green fluorescence ratio, indicating a significant loss of mitochondrial membrane potential (Supplementary Figure S1D,E). Consistent with these functional deficits, the mtDNA/nDNA ratio was also significantly reduced following BXA treatment (Figure 1L). Taken together, these findings demonstrate that BXA treatment progressively impairs mitochondrial function, affecting calcium homeostasis, membrane integrity, and mitochondrial genome maintenance.

### 2.2. Live-Cell Imaging Reveals Dynamic Mitochondrial Morphological Changes

**Figure 2:**
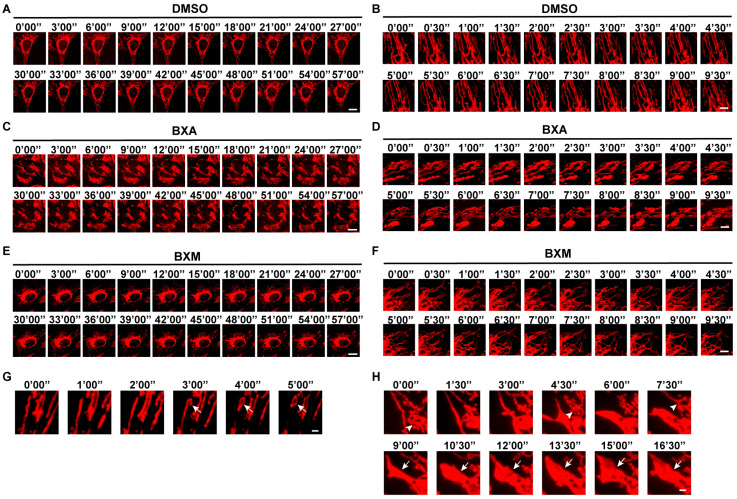
Real-time live-cell imaging reveals baloxavir-induced mitochondrial morphological dynamics. Mitochondria were labeled with COX8-mScarlet. (**A**–**F**) Live-cell time-lapse imaging of mitochondrial morphology dynamics in U2OS cells treated with DMSO (**A**,**B**), 10 μM BXA (**C**,**D**), or 10 μM BXM (**E**,**F**). (**B**,**D**,**F**) show magnified views in a shorter term (from cells different from (**A**,**C**,**E**)). (**G**) Representative images showing annular mitochondrial structures (mitochondrial “donuts”) (arrows) following BXA treatment. (**H**) Examples of mitochondrial aberrant branching (top panel, arrowheads) and swelling (megamitochondria) (bottom panel, arrows) observed after BXA exposure. Scale bars: 20 μm (**A**,**C**,**E**), 5 μm (**B**,**D**,**F**), 2 μm (**G**,**H**).

To delineate the temporal dynamics of mitochondrial changes, we performed live-cell time-lapse imaging during the first few hours of drug exposure. Vehicle control cells maintained elongated, interconnected mitochondrial networks with normal fission–fusion dynamics (Figure 2A,B and Figure S2A,B; Videos S1 and S2). In contrast, BXA exposure induced various morphological abnormalities (Figure 2C,D and Figure S2C,D; Videos S3 and S4), including annular structures (mitochondrial “donuts”), pronounced swelling (megamitochondria), and aberrant branching [35,36] (Figure 2G,H). These heterogeneous morphologies may represent different stages of progressive mitochondrial damage following BXA treatment. BXM again failed to induce noticeable morphological abnormalities (Figure 2E,F and Figure S2E,F; Videos S5 and S6).

### 2.3. BXA Also Induces Autophagy

**Figure 3:**
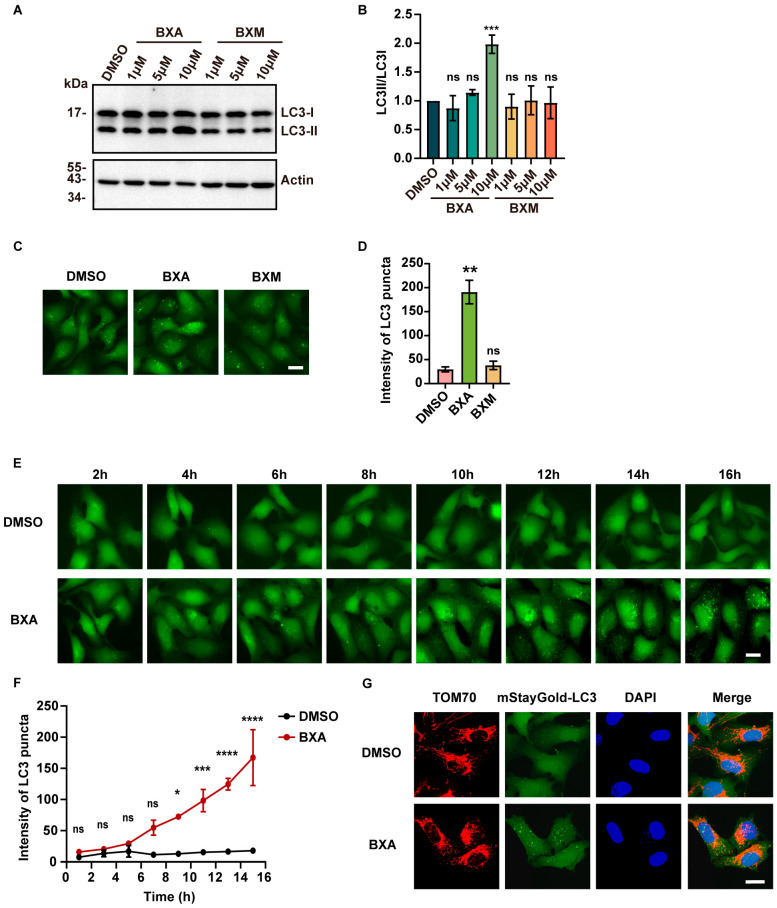
Baloxavir-induced autophagy is independent of mitochondrial abnormalities. (**A**) Western blot analysis of LC3-I and LC3-II levels in U2OS cells treated with BXA or BXM for 16 h. (**B**) Quantification of the LC3-II/LC3-I ratio from (**A**). (**C**) Representative images of autophagy in U2OS cells upon drug treatments, detected using mStayGold-LC3 as an autophagosome marker. (**D**) Quantitative analysis of LC3 puncta fluorescence intensity corresponding to (**C**). (**E**) Live-cell time-lapse imaging of mStayGold-LC3 in U2OS cells showing autophagy dynamics over 16 h following treatment with 10 μM BXA. (**F**) Quantification of LC3 puncta intensity over 16 h from (**E**). Data points represent the average fluorescence intensity measured at 2-h intervals. (**G**) Representative images showing the localization of mStayGold-LC3 (autophagosomes) and TOM70 (mitochondria) at 16 h after treatment with 10 μM BXA. Scale bar: 20 μm (**C**,**E**,**G**). Data are presented as mean ± SEM from three independent experiments. ns, not significant; * *p* < 0.05, ** *p* < 0.01, *** *p* < 0.001, **** *p* < 0.0001 vs. control group.

During high-content screening, we noted that BXA also induced moderate autophagy in U2OS cells, as detected using the autophagic marker mStayGold-LC3. Western blot analysis showed that BXA (10 μM) increased the LC3II/LC3I ratio after 16 h of treatment, confirming autophagy activation (Figure 3A,B). In contrast, BXM did not elevate LC3II levels. Consistent with biochemical results, BXA treatment modestly enhanced LC3 puncta formation (Figure 3C,D). Temporal analysis further demonstrated that BXA induced progressive accumulation of LC3 puncta starting around 8 h post-treatment (Figure 3E,F; Videos S7 and S8). Of note, autophagy induction lagged behind mitochondrial changes, which occurred within hours after treatment. Since mitochondrial damage can trigger mitophagy, we hypothesized that BXA-induced autophagy might represent mitophagy. However, weak colocalization between TOM70 (a mitochondrial outer membrane protein) [37,38] and LC3 puncta indicated that autophagy was not specifically targeted to mitochondria [39,40] (Figure 3G). Together with the delayed onset of autophagy relative to mitochondrial abnormalities, we conclude that BXA induces mitochondrial damage and autophagy through distinct pathways.

Next, we would focus on mitochondrial toxicity for two reasons: first, baloxavir-induced autophagy was less pronounced than mitochondrial alterations; second, autophagosomes did not encapsulate or surround fragmented mitochondria, indicating that autophagy induction is independent of mitochondrial damage.

### 2.4. BXA Treatment Leads to Cell Cycle Arrest and Apoptosis

**Figure 4:**
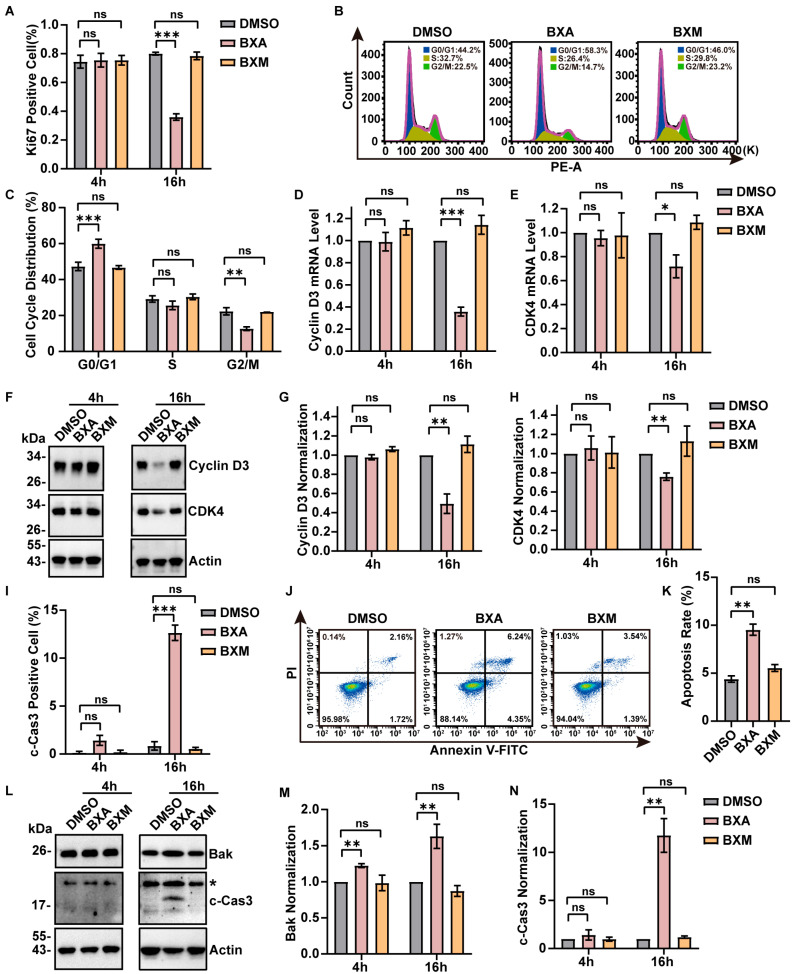
BXA treatment induces cell cycle arrest and apoptosis. (**A**) Quantification of Ki67-positive U2OS cells following treatment with BXA or BXM (10 μM, 4 h or 16 h). (**B**) Flow cytometry analysis of cell cycle distribution in U2OS cells after 16 h of drug treatments. (**C**) Quantification of the percentage of cells in G0/G1, S, and G2/M phases from (**B**). (**D**,**E**) qPCR analysis showing mRNA levels of cyclin D3 (**D**) and CDK4 (**E**) in U2OS cells after 4 h or 16 h of drug treatments. (**F**) Western blot analysis of cyclin D3 and CDK4 protein expression in U2OS cells treated as indicated. (**G**,**H**) Quantification of cyclin D3 (**G**) and CDK4 (**H**) protein levels from (**F**). (**I**) Quantification of cleaved caspase-3-positive cells following treatment with BXA or BXM (10 μM, 4 h or 16 h). (**J**) Apoptosis detection by flow cytometry using Annexin V staining in U2OS cells after 16 h drug exposure. (**K**) Quantification of apoptotic cells from (**J**). (**L**) Western blot analysis of Bak and cleaved caspase-3 protein levels in U2OS cells treated with 10 μM BXA or BXM for the indicated durations. * Indicates non-specific bands. (**M**,**N**) Quantification of Bak (**M**) and cleaved caspase-3 (**N**) protein levels from (**L**). Data are presented as mean ± SEM from three independent experiments. ns, not significant; * *p* < 0.05, ** *p* < 0.01, *** *p* < 0.001 vs. control group.

We observed reduced cell numbers after BXA treatment compared with the control and BXM groups. Staining with the proliferation marker Ki67 confirmed that BXA (10 μM), but not BXM, substantially inhibited cellular proliferation (Figure 4A and Figure S3A). Flow cytometric analysis indicated that BXA treatment induced cell cycle arrest at the G0/G1 checkpoint (Figure 4B,C). Correspondingly, key G0/G1 regulators, cyclin D3 and CDK4 [41], were significantly downregulated at both transcriptional and protein levels after 16 h- but not at 4 h-post-treatment (Figure 4D–H), providing a molecular basis for proliferation arrest. Furthermore, immunofluorescence analysis showed that BXA treatment (16 h) significantly increased the proportion of cleaved caspase-3-positive cells, indicating apoptosis (Figure 4I and Figure S3B). Flow cytometry with Annexin V staining confirmed that BXA, but not BXM, effectively induced apoptosis (Figure 4J,K). Given the early mitochondrial abnormalities (observed 2–6 h after treatment), we hypothesized that these initial mitochondrial events lead to subsequent cell cycle arrest and apoptosis (evident at 16 h). Indeed, we detected mildly elevated Bak expression after BXA treatment (10 μM) (Figure 4L,M), which temporally correlated with increased cleaved caspase-3 levels (Figure 4L,N), suggesting involvement of the Bak-caspase-3 axis in BXA-induced apoptosis.

### 2.5. N-Acetylcysteine Alleviates BXA-Induced Mitochondrial Fragmentation and the Decreased Expression of Cyclin D3

**Figure 5:**
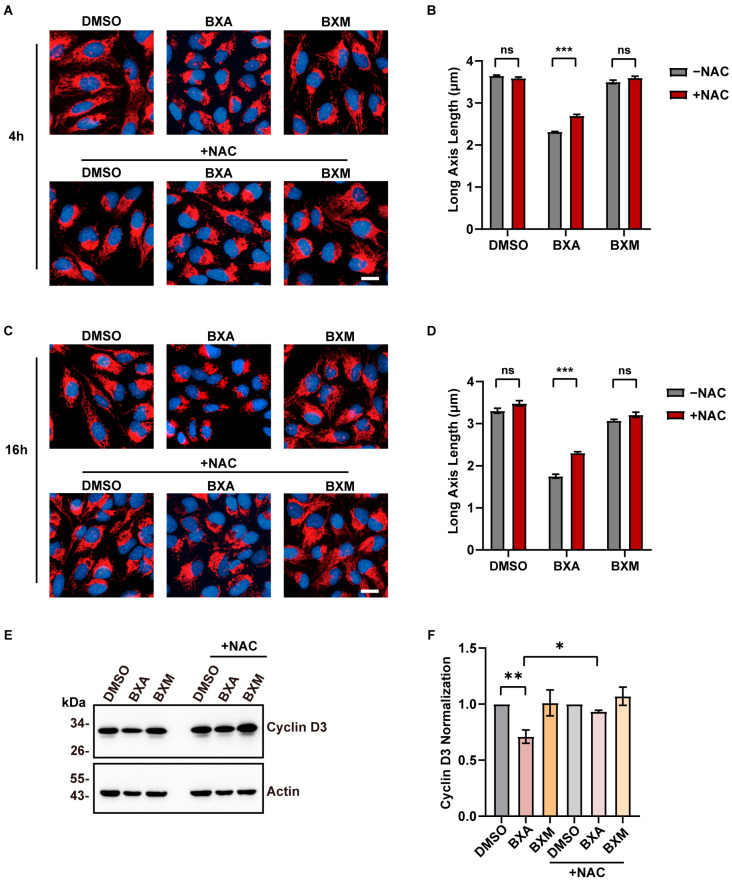
NAC treatment partially alleviates BXA-induced mitochondrial fragmentation and the downregulation of cyclin D3. (**A**) Representative images of mitochondrial morphology in U2OS cells treated with the indicated drugs alone or in combination with NAC for 4 h. Mitochondria were stained with TMRM (red), and nuclei were stained with Hoechst (blue). (**B**) Quantification of the mitochondrial long-axis length corresponding to the treatment groups in (**A**). (**C**) Representative images of mitochondrial morphology in U2OS cells treated with the indicated drugs alone or in combination with NAC for 16 h. Mitochondria were stained with TMRM (red), and nuclei were stained with Hoechst (blue). (**D**) Quantification of the mitochondrial long-axis length corresponding to the treatment groups in (**C**). (**E**) Western blot analysis of cyclin D3 protein level in U2OS cells treated with the indicated drugs alone or in combination with NAC for 16 h. (**F**) Quantification of cyclin D3 from three independent experiments corresponding to (**E**). Scale bar: 20 μm (**A**,**C**). Data are presented as mean ± SEM from three independent experiments. ns, not significant; * *p* < 0.05, ** *p* < 0.01, *** *p* < 0.001 vs. control group.

As a broad-spectrum antioxidant, N-acetylcysteine (NAC) plays an important role in protecting mitochondria from oxidative damage. To investigate its potential protective effects, U2OS cells were pretreated with 5 mM NAC for 2 h, followed by co-treatment with BXA or BXM. NAC co-treatment alleviated BXA-induced mitochondrial morphological abnormalities at both 4 h and 16 h (Figure 5A–D). Consistently, after 16 h of co-treatment, the expression of cyclin D3 was higher in cells treated with NAC and BXA compared to those treated with BXA alone (Figure 5E,F). Together, these results indicate that NAC treatment partially rescues BXA-induced mitochondrial morphological abnormalities and the decreased expression level of cyclin D3.

### 2.6. BXA Induces Liver Toxicity In Vivo

**Figure 6:**
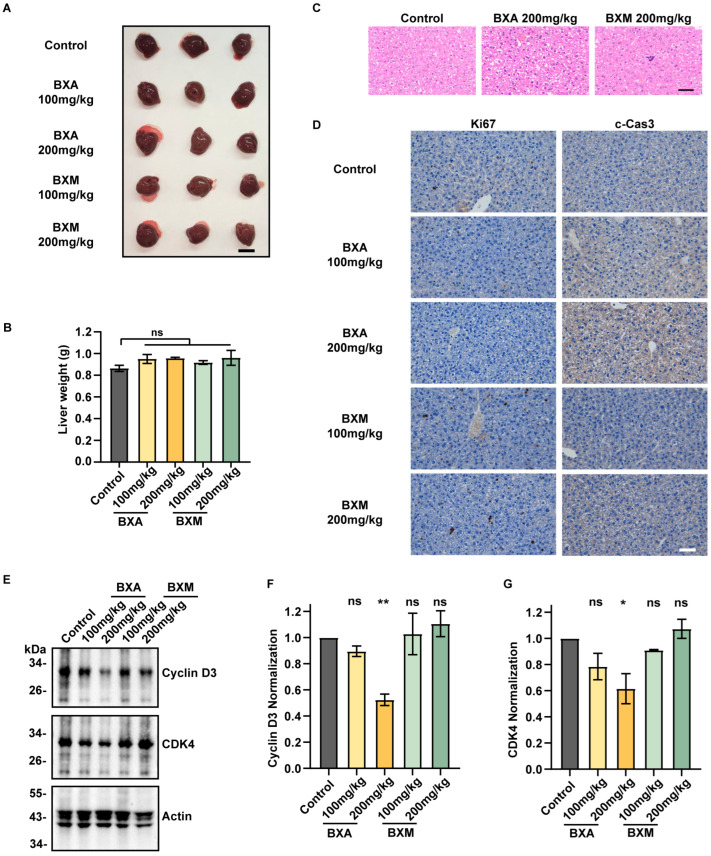
BXA induces hepatocyte cell cycle perturbation in vivo. (**A**) Images of mouse livers collected 24 h after administration of BXA or BXM at the indicated concentrations. Scale bar: 1 cm. (**B**) Liver weight analysis after drug treatment (*n* = 3 mice per group). (**C**) H&E staining of liver tissues. Scale bar: 50 μm. (**D**) Immunohistochemical staining of mouse liver sections for Ki67 (proliferation marker) and cleaved caspase-3 (apoptosis marker) after 24 h drug exposure. Scale bar: 50 μm. (**E**) Western blot analysis of cyclin D3 and CDK4 expression in mouse liver tissues following drug administration. (**F**,**G**) Quantification of cyclin D3 (**F**) and CDK4 (**G**) protein levels from (**E**). Data are presented as mean ± SEM from three independent experiments. ns, not significant; * *p* < 0.05, ** *p* < 0.01 vs. control group.

To assess the clinical relevance of our findings, we evaluated the in vivo toxicity of BXA and its prodrug, BXM. Mice were treated with BXA or BXM for 24 h. Throughout this period, no apparent health impairments were observed (Supplementary Figure S4A,B). The liver, being the primary site where BXM is metabolized to BXA, was subsequently analyzed. Although liver morphology and weight remained unchanged across treatment groups (Figure 6A,B), histopathological examination via H&E staining revealed distinct differences at the tissue level. Liver sections from the BXA (200 mg/kg) treatment group displayed pronounced hepatocellular edema with vacuolated, pale-stained cytoplasm, indicating liver injury (Figure 6C). Furthermore, immunohistochemical analysis revealed dose-dependent reductions in Ki67 and increases in cleaved caspase-3 in hepatic tissues following BXA administration (Figure 6D). In contrast, BXM exerted markedly milder effects. Western blot analysis further confirmed dose-dependent decreases in cyclin D3 and CDK4 expression in BXA-treated, but not BXM-treated, animals (Figure 6E–G), supporting in vivo cell cycle disruption consistent with our in vitro observations. These data provide a mechanistic explanation for baloxavir-associated adverse effects, such as liver dysfunction [28]. Collectively, these in vivo results demonstrate that BXA induces cellular toxicity through mitochondrial disruption and cell cycle perturbation.

## 3. Discussion

Overall, this study delineates the morphological and dynamic alterations in mitochondria and autophagy following treatment with baloxavir acid (BXA) and its prodrug, baloxavir marboxil (BXM). Spatiotemporal analysis reveals that BXA-induced mitochondrial morphological damage and autophagy likely represent independent cellular pathways. Notably, BXA exerts predominant mitochondrial toxicity, which subsequently drives cell cycle arrest mediated by downregulation of cyclin D3 and CDK4, and induces apoptosis via the Bak-caspase-3 axis, as demonstrated both in vitro and in vivo. Given that BXA’s intended target is the influenza viral PA protein, the observation that it elicits organelle-level effects at micromolar concentrations (in the absence of PA protein), substantially higher than its nanomolar-range anti-influenza EC_50_ [14,42], indicates the involvement of off-target mechanisms. The peak plasma concentration of baloxavir acid in humans is approximately 0.2–0.4 μM [43,44]. It is well recognized that drug concentrations in metabolic tissues, such as the liver, are often 10-fold or higher than those in plasma [45,46,47]. Therefore, the local concentration of BXA in the liver could reasonably reach 5–10 μM, which aligns with the concentration range used in the present study to induce localized cytotoxicity. Although the host factors responsible for BXA-triggered mitochondrial dysfunction remain unidentified, our findings provide important mechanistic insights into the drug’s cytotoxicity, which may be relevant to its potential clinical adverse effects.

A key distinction emerged from the comparison with BXM, the prodrug, which exhibited markedly attenuated cytotoxicity relative to BXA. The administration of the less toxic prodrug BXM, followed by its metabolic conversion, prevents the acute cytotoxicity associated with direct BXA exposure. Importantly, BXA, which is generated from BXM in primary metabolic tissues (e.g., the liver and intestine), enters the systemic circulation and undergoes extensive tissue distribution (as reflected by its large volume of distribution). This process thereby lowers its local concentration at the site of formation and reduces the potential for organ-specific toxicity. Nevertheless, the most frequently reported adverse reactions to baloxavir involve the gastrointestinal tract and liver, consistent with residual cytotoxic activity at the primary metabolic sites. Thus, beyond enhancing bioavailability, the prodrug strategy confers a significant therapeutic advantage by substantially reducing cytotoxicity.

In summary, this study establishes a link between baloxavir-induced cytotoxicity and its adverse clinical effects. Further investigation is needed to elucidate the detailed underlying mechanisms and explore strategies to mitigate these adverse outcomes.

## 4. Materials and Methods

### 4.1. Cell Culture

Human HeLa cells (ATCC CCL-2) were maintained in Dulbecco’s Modified Eagle Medium (DMEM) supplemented with 10% fetal bovine serum (FBS). Human U2OS cells (ATCC HTB-96) were cultured in McCoy’s 5A medium containing 10% FBS. Both cell lines were incubated at 37 °C in a humidified atmosphere with 5% CO_2_.

### 4.2. Antibodies and Reagents

Primary antibodies employed for immunofluorescence included: TOM70 (Proteintech, Rosemont, IL, USA, 14528-1-AP), TIM50 (Proteintech, 22229-1-AP), Ki67 (8D5) (Cell Signaling Technology, Danvers, MA, USA, #9449), and Cleaved Caspase-3 (Asp175) (Cell Signaling Technology, #9661). DAPI (Merck, Darmstadt, Hesse, Germany, D9542) was used to stain cell nuclei.

For Western blot analysis, the following primary antibodies were utilized: β-actin (Biodragon, Suzhou, Jiangsu, China, B1029; Selleck, Houston, TX, USA, F0082), LC3B (D11) (Cell Signaling Technology, #3868), Bak (D2D3) (Cell Signaling Technology, #6947), Cleaved Caspase-3 (Asp175) (Cell Signaling Technology, #9661), Cyclin D3 (DCS22) (Cell Signaling Technology, #2936), and CDK4 (Proteintech, 11026-1-AP).

Pharmaceutical compounds included baloxavir acid (MedChemExpress, Monmouth Junction, NJ, USA, HY-109025A), baloxavir marboxil (MedChemExpress, HY-109025) and N-Acetylcysteine (MedChemExpress, HY-B0215). Live-cell imaging experiments utilized the following fluorescent dyes: Hoechst 33342 (10 μg/mL, Invitrogen, Carlsbad, CA, USA, H3570), tetramethylrhodamine (TMRM) (MedChemExpress, HY-D0984). For the assessment of mitochondrial membrane potential and calcium levels, the following fluorescent dyes were employed: JC-1 (MedChemExpress, HY-15534) and Rhod-2 AM (Abcam, Cambridge, England, UK, Ab142780).

### 4.3. Plasmid Construction and Validation

A mitochondria targeting clone, COX8-mScarlet, was generated by fusing the mitochondrial targeting sequence of cytochrome c oxidase subunit 8 (COX8) to the N-terminus of the fluorescent protein mScarlet, and the fusion sequence was subsequently cloned into the pLVX lentiviral expression vector. For autophagy monitoring, the coding sequence of the fluorescent protein mStayGold was fused to the N-terminus of the LC3 sequence, and the fusion sequence was inserted into the pLVX lentiviral vector.

All recombinant plasmids were verified by sequencing.

### 4.4. Immunofluorescence

U2OS cells were seeded onto glass coverslips in 24-well plates and treated with either dimethyl sulfoxide (DMSO), baloxavir acid or baloxavir marboxil at specified concentrations and time points. Following treatment, cells were fixed with prewarmed 4% paraformaldehyde (PFA) at 37 °C for 10 min, then permeabilized using 0.2% Triton X-100 in phosphate-buffered saline (PBS) for 10 min. Non-specific binding was blocked through incubation with 3% bovine serum albumin (BSA) in PBS for 30 min at room temperature. Cells were subsequently incubated with primary antibodies at 37 °C for 2 h, followed by three PBS washes and incubation with appropriate fluorescence-conjugated secondary antibodies at 37 °C for 1 h. Nuclear staining was performed with 4′,6-diamidino-2-phenylindole (DAPI), and coverslips were mounted using ProLong™ Diamond Antifade Mountant (Invitrogen, P36970). Images were acquired using a Leica Stellaris 5 confocal microscope.

### 4.5. Western Blot

Cellular proteins were extracted using SDS-containing lysis buffer. Cell lysates were boiled and separated by sodium dodecyl sulfate-polyacrylamide gel electrophoresis (SDS-PAGE), followed by electrotransfer onto nitrocellulose membranes.

Membranes were probed with primary antibodies for 2 h at room temperature, then incubated with horseradish peroxidase-conjugated secondary antibodies for 1 h at room temperature. Immunoreactive bands were visualized using an enhanced chemiluminescence (ECL) detection kit (Invitrogen, 34580) and captured with a Tanon 5800 chemiluminescent imaging system (Shanghai, China). Experiments were performed in triplicate, and protein quantification was conducted using ImageJ software 2.16.0.

### 4.6. Live-Cell Time-Lapse Imaging

For live-cell mitochondria imaging, U2OS cells were pre-incubated with 250 nM TMRM for 1 h, then co-treated with either 10 μM baloxavir acid or baloxavir marboxil and 250 nM TMRM. Cells were immediately transferred to the CellDiscoverer 7 (CD7) system (Zeiss) and imaged at 30-s intervals for 4 h at 37 °C. Alternatively, the U2OS COX8-mScarlet stable cell line was used by capturing images at 30-s intervals for 4 h immediately following drug treatment.

For live-cell autophagy imaging, U2OS mStayGold-LC3 stable cells were imaged at 10-min intervals for 16 h following drug treatment. The average fluorescence intensity of LC3 puncta was calculated every 2 h to determine autophagic level.

### 4.7. Image Analysis and Morphometric Quantification

All live-cell images underwent automated analysis using CellProfiler software for nuclear segmentation, mitochondrial morphology assessment, or LC3 puncta intensity quantification. Statistical analysis of morphometric parameters and fluorescence intensity measurements was subsequently performed.

### 4.8. JC-1 Staining

U2OS cells were seeded in 48-well plates and treated with the indicated compounds for 4 or 16 h. After treatment, cells were gently rinsed twice with fresh culture medium. Subsequently, cells were incubated with 2 μM JC-1 staining solution, prepared by diluting a 2 mM JC-1 stock solution 1:1000 in Opti-MEM medium, for 20 min at 37 °C in a cell culture incubator protected from light. Fluorescent images were captured using an Evos FL Auto microscope (ThermoFisher Scientific, Wilmington, DE, USA). The obtained images were analyzed with ImageJ software, and the ratio of red to green fluorescence intensity was calculated for individual cells.

### 4.9. Rhod-2 AM Staining

U2OS cells were treated with indicated compounds for 4 h or 16 h. Then cells were incubated with Rhod-2 AM (5 μM) in HBSS at 37 °C for 25 min. After rinsing cells three times with DPBS, the fluorescent images were captured using an Evos FL Auto microscope (ThermoFisher Scientific) at 20× magnification. Use CellProfiler to calculate the mean fluorescence intensity of each cell.

### 4.10. Mitochondrial DNA Quantification

Total genomic DNA was extracted from U2OS cells at 4 h or 16 h post-treatment using the TIANamp Genomic DNA Kit (TIANGEN, Beijing, China, DP304). Quantitative real-time PCR (qPCR) was performed using the HiScript II One Step qRT-PCR SYBR Green Kit (Vazyme, Nanjing, Jiangsu, China, Q221-01) according to manufacturer protocols.

The mitochondrial NADH dehydrogenase 4 (MT-ND4) gene served as the mitochondrial DNA target, while β2-microglobulin (β2M) was used as the nuclear DNA reference gene. Relative mitochondrial DNA copy number was calculated using the 2^−ΔΔCT^ method, where ΔCT represents the difference between average cycle threshold (CT) values of the target and reference genes.

Primer sequences were as follows:

- Human MT-ND4 Forward: 5′-CACCCAAGAACAGGGTTTGT-3′
- Human MT-ND4 Reverse: 5′-TGGCCATGGGTATGTTGTTA-3′
- Human β2M Forward: 5′-TGCTGTCTCCATGTTTGATGTATCT-3′
- Human β2M Reverse: 5′-TCTCTGCTCCCCACCTCTAAGT-3′

### 4.11. Quantitative Real-Time PCR Analysis

Total RNA was isolated using RNAfast200 reagent (Fastagen, Langfang, Hebei, China, Cat. #220011) at 4 h or 16 h post drug treatment. Quantitative PCR was performed using the HiScript II One Step qRT-PCR SYBR Green Kit (Vazyme, Nanjing, Jiangsu, China, Q221-01), with glyceraldehyde-3-phosphate dehydrogenase (GAPDH) serving as the housekeeping gene control.

Primer sequences utilized were:

- Human GAPDH Forward: 5′-GTCTCCTCTGACTTCAACAGCG-3′
- Human GAPDH Reverse: 5′-ACCACCCTGTTGCTGTAGCCAA-3′
- Human Cyclin D3 Forward: 5′-AGATCAAGCCGCACATGCGGAA-3′
- Human Cyclin D3 Reverse: 5′-ACGCAAGACAGGTAGCGATCCA-3′
- Human CDK4 Forward: 5′-CCATCAGCACAGTTCGTGAGGT-3′
- Human CDK4 Reverse: 5′-TCAGTTCGGGATGTGGCACAGA-3′

### 4.12. Cell Cycle Analysis

U2OS cells were seeded in 6-well plates at 70% confluence and treated with 10 μM baloxavir acid or baloxavir marboxil for 16 h. Then cells were harvested, washed twice with PBS, and fixed overnight at 4 °C with ice-cold 70% ethanol.

Fixed cells were washed twice with PBS and incubated with RNase A (100 μg/mL) at 37 °C for 20 min to eliminate RNA interference. Subsequently, cells were stained with propidium iodide (PI) (10 μg/mL) in the dark for 30 min. Cell cycle distribution was analyzed using a CytoFLEX flow cytometer (Beckman Coulter, Brea, CA, USA), and data processing was performed using FlowJo software 10.8.1 (BD Biosciences, Milpitas, CA, USA).

### 4.13. Apoptosis Quantification

Apoptotic cell death was assessed using the Annexin V-FITC/PI Apoptosis Detection Kit (BD Biosciences, Cat# 556547). Following 16-h drug treatment, U2OS cells were trypsinized, harvested, and washed once with ice-cold PBS before resuspension in 300 μL of 1× binding buffer.

Cells were incubated with 5 μL Annexin V-FITC in the dark at room temperature for 15 min, followed by the addition of 5 μL propidium iodide staining solution. The final volume was adjusted to 500 μL with 1× binding buffer prior to flow cytometric analysis. Appropriate unstained and single-stained controls were processed in parallel for compensation purposes.

### 4.14. N-Acetylcysteine Rescue Assay

U2OS cells were seeded in 96-well or 12-well plates and pretreated with 5 mM N-acetylcysteine (NAC) for 2 h. Following pretreatment, the medium was removed, and cells were incubated with fresh culture medium containing 10 μM baloxavir acid or baloxavir marboxil, with or without 5 mM NAC, for an additional 4 or 16 h. Then measure mitochondrial morphology and analyze cyclin D3 expression.

### 4.15. In Vivo Hepatotoxicity Assessment

Ten-week-old female BALB/c mice were obtained from Peking University Health Science Center and housed under standard animal facility conditions. Following a one-week acclimatization period, mice were randomly allocated into five experimental groups (*n* = 3 per group) to evaluate drug-induced hepatotoxicity. All compounds were freshly suspended in 0.5% methylcellulose solution immediately prior to administration and delivered via oral gavage in a standardized volume of 500 μL per mouse. At 24 h post drug administration, mice were euthanized with institutional animal care guidelines. Liver tissues were immediately harvested for subsequent H&E staining, immunohistochemistry examination and protein expression analysis. All mice experiments were approved by the Institutional Animal Care and Use Committee (IACUC) of the Chinese Academy of Medical Sciences and Peking Union Medical College (CAMS & PUMC), and were performed in accordance with relevant guidelines and regulations.

### 4.16. Statistical Analysis

All statistical analyses were conducted using GraphPad Prism 8.0 software. Experimental data are presented as means ± standard error of the mean (SEM). Statistical comparisons between experimental groups were performed using unpaired two-tailed Student’s *t*-tests. All experiments were performed with appropriate biological replicates as specified for each assay.

## 5. Conclusions

Our work presents the first evidence that baloxavir acid (BXA), the active metabolite of baloxavir marboxil (BXM), induces significant mitochondrial abnormalities as an off-target effect, leading to cell cycle arrest and apoptosis. This work addresses a critical gap in understanding the cellular basis of clinical side effects associated with baloxavir.

## Data Availability

The original contributions presented in this study are included in the article. Further inquiries can be directed to the corresponding author.

## Supplementary Materials

The following supporting information can be downloaded at: https://www.mdpi.com/article/10.3390/ijms27072967/s1.

## Abbreviations

The following abbreviations are used in this manuscript:

BXA	Baloxavir acid
BXM	Baloxavir marboxil
NAC	N-acetylcysteine
PA	Polymerase acidic
TMRM	Tetramethylrhodamine
