# Supplementary material for: Baloxavir Acid-Induced Mitochondrial Toxicity and Cell Cycle Arrest Contribute to Its Adverse Effects
Source: Int J Mol Sci. 2026 Mar 25;27(7):2967. doi: 10.3390/ijms27072967 (PMC13073450; doi:10.3390/ijms27072967)
Supplement: Supplementary file 1 [file ijms-27-02967-s001.zip › supplementary-tracked.pdf]

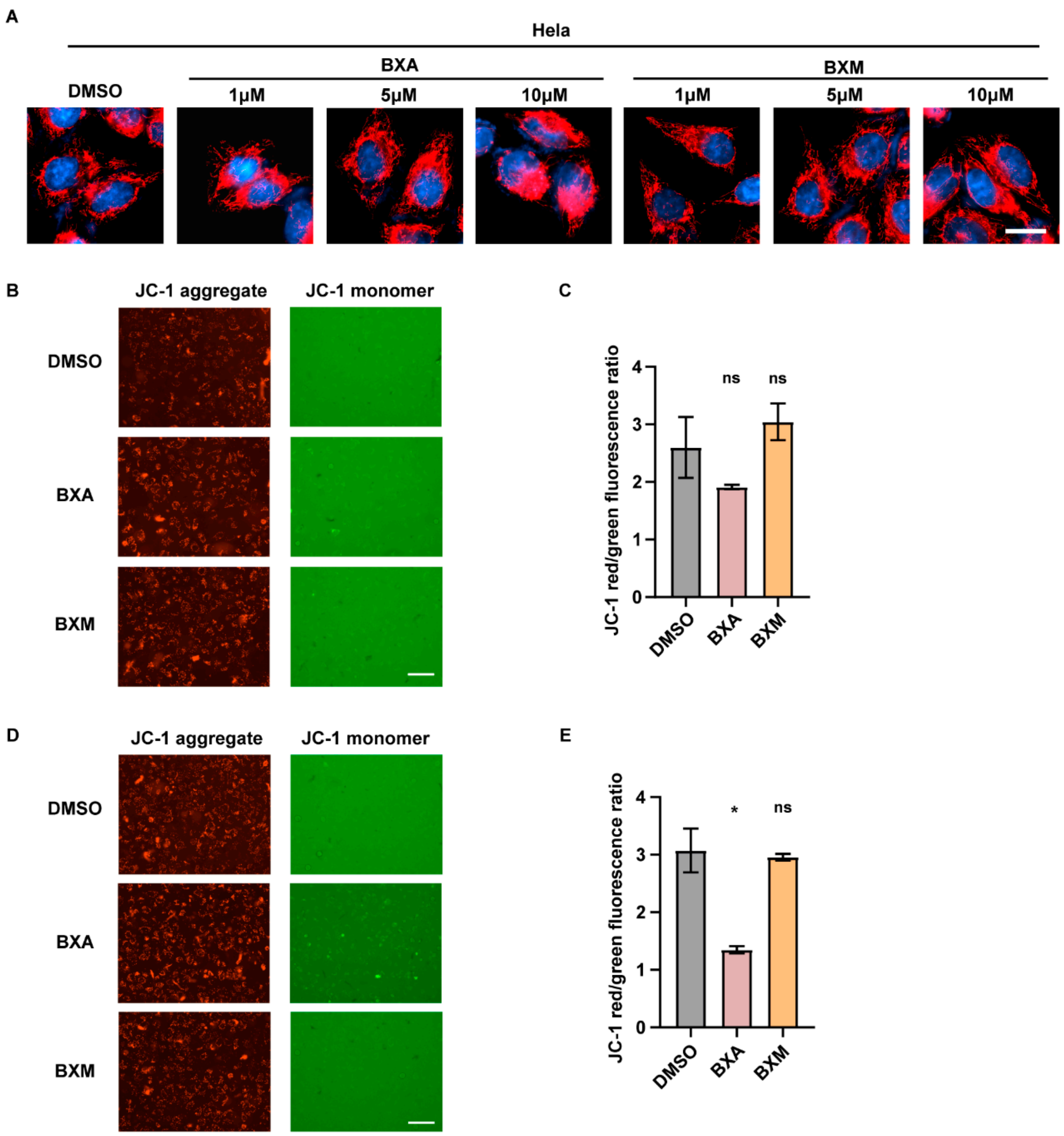

Figure S1. Baloxavir induces mitochondrial morphological abnormalities and functional damage. **(A)** Representative images showing mitochondrial morphology in HeLa cells following 24-hour exposure to BXA or BXM. Mitochondria were stained with TMRM (red) and nuclei with Hoechst (blue). Scale bar: 20 μm. **(B)** Representative images of JC-1 staining after 4 h of drug treatment. Scale bar: 200 μm. **(C)** Quantification of JC-1 red/green fluorescence ratio from (**B**). (**D**) Representative images of JC-1 staining after 16 h of drug treatment. Scale bar: 200 μm. (**E**) Quantification of JC-1 red/green fluorescence ratio from (**D**). Data are presented as mean ± SEM from three independent experiments. ns, not significant; $^*P < 0.05$, $^{**}P < 0.01$, $^{***}P < 0.001$ vs. control group.

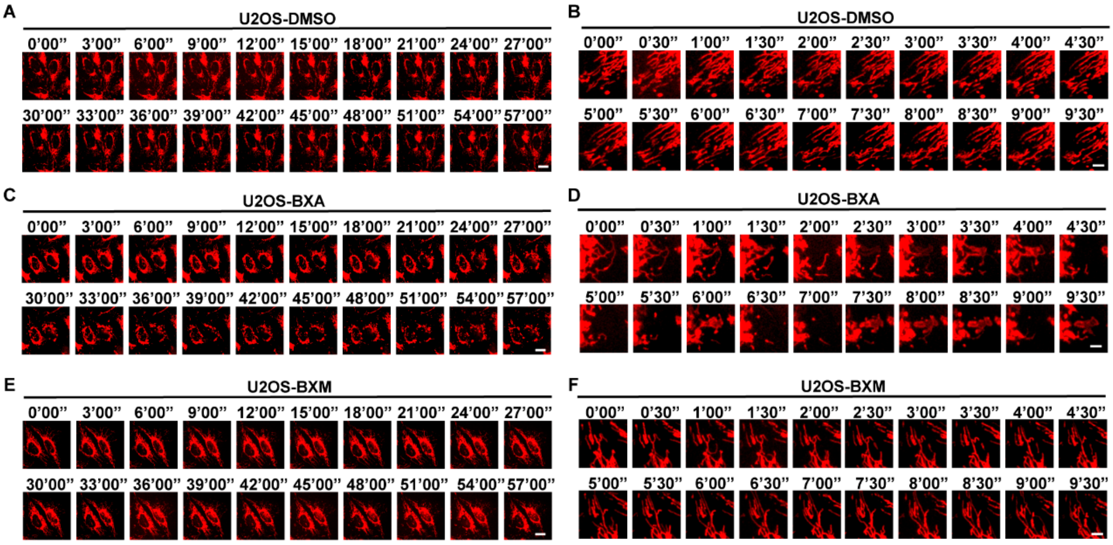

Figure S2. Real-time live-cell imaging reveals baloxavir-induced mitochondrial morphological dynamics. Mitochondria were labeled with TMRM (red). (**A-F)** Live-cell time-lapse imaging of mitochondrial morphology dynamics in U2OS cells treated with DMSO (**A** and **B**), 10 μM BXA (**C** and **D**), or 10 μM BXM (**E** and **F**). (**B**, **D** and **F**) show magnified views in a shorter term (from cells different from **A**, **C** and **E**). Scale bars: 20 μm (**A**, **C**, and **E**), 5 μm (**B**, **D** and **F**).

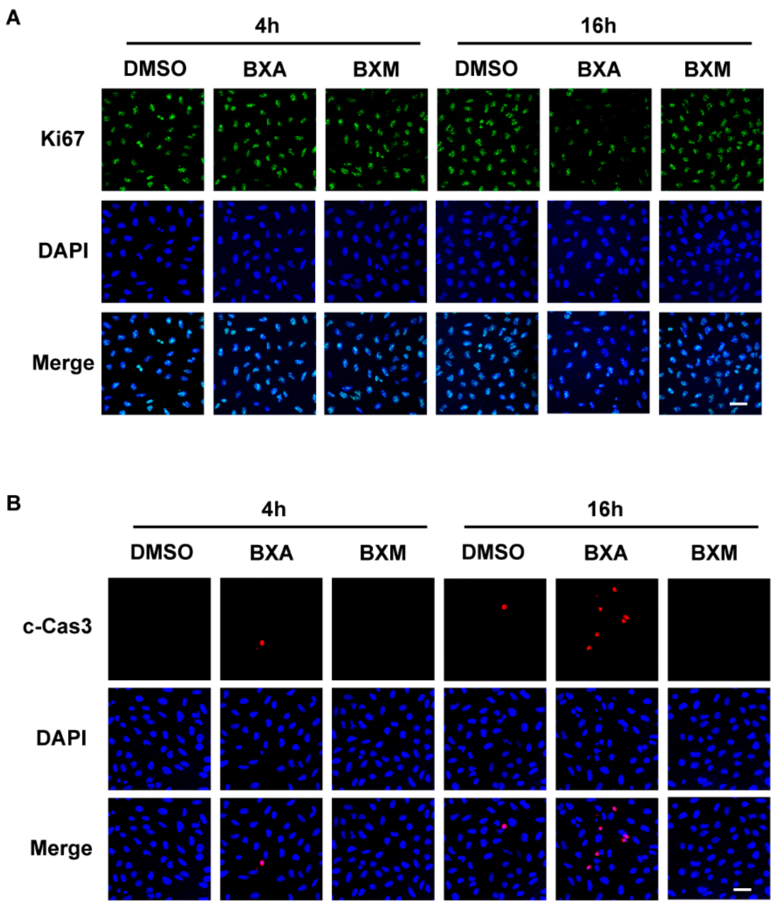

Figure S3. BXA inhibits proliferation and promotes apoptosis in U2OS cells. **(A)** Immunofluorescence staining of Ki67 in U2OS cells treated with 10 μM BXA or BXM for 4 h or 16 h. (**B)** Immunofluorescence staining of cleaved caspase-3 in U2OS cells treated with 10 μM BXA or BXM for 4 h or 16 h. Scale bar: 50 μm (**A** and **B**).

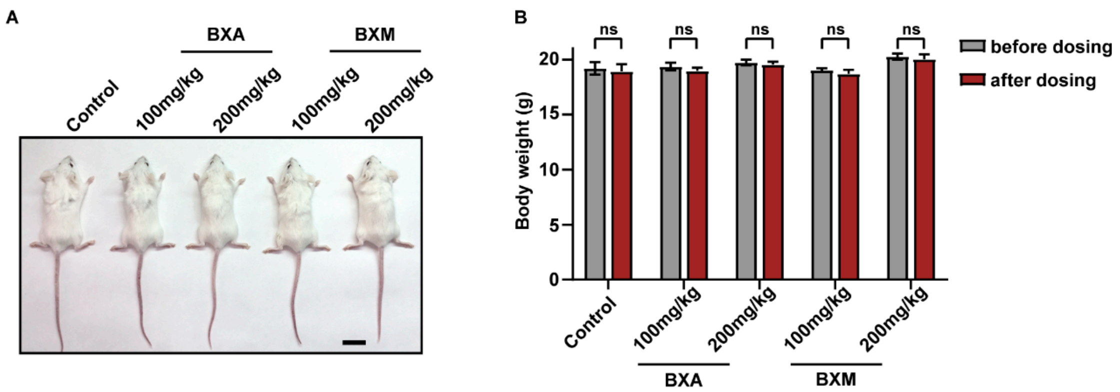

Figure S4. Evaluation of mouse health status following drug administration. **(A)** Representative photographs showing the overall physical appearance of mice from each treatment group. Scale bar: 2 cm. (**B)** Body weight of mice measured before and after the 24-hour drug treatment period. Data are presented as mean ± SEM (n = 3 mice per group). ns, not significant; *P < 0.05, **P < 0.01, ***P < 0.001 vs. control group.

Video 1. One-hour time-lapse (interval=30 sec/slice) imaging of U2OS cells expressing COX8-mScarlet (red) under DMSO treatment.

Video 2. One-hour time-lapse (interval=30 sec/slice) imaging of U2OS cells stained with TMRM (red) under DMSO treatment.

Video 3. One-hour time-lapse (interval=30 sec/slice) imaging of U2OS cells expressing COX8-mScarlet (red) under BXA treatment.

Video 4. One-hour time-lapse (interval=30 sec/slice) imaging of U2OS cells stained with TMRM (red) under BXA treatment.

Video 5. One-hour time-lapse (interval=30 sec/slice) imaging of U2OS cells expressing COX8-mScarlet (red) under BXM treatment.

Video 6. One-hour time-lapse (interval=30 sec/slice) imaging of U2OS cells stained with TMRM (red) under BXM treatment.

Video 7. Sixteen-hour time-lapse (interval=10 min/slice) imaging of U2OS cells expressing mStayGold-LC3 (green) under DMSO treatment.

Video 8. Sixteen-hour time-lapse (interval=10 min/slice) imaging of U2OS cells expressing mStayGold-LC3 (green) under BXA treatment.
