# Supplementary figures and images for: Baloxavir Acid-Induced Mitochondrial Toxicity and Cell Cycle Arrest Contribute to Its Adverse Effects
Source: Int J Mol Sci. 2026 Mar 25;27(7):2967. doi: 10.3390/ijms27072967 (PMC13073450; doi:10.3390/ijms27072967)

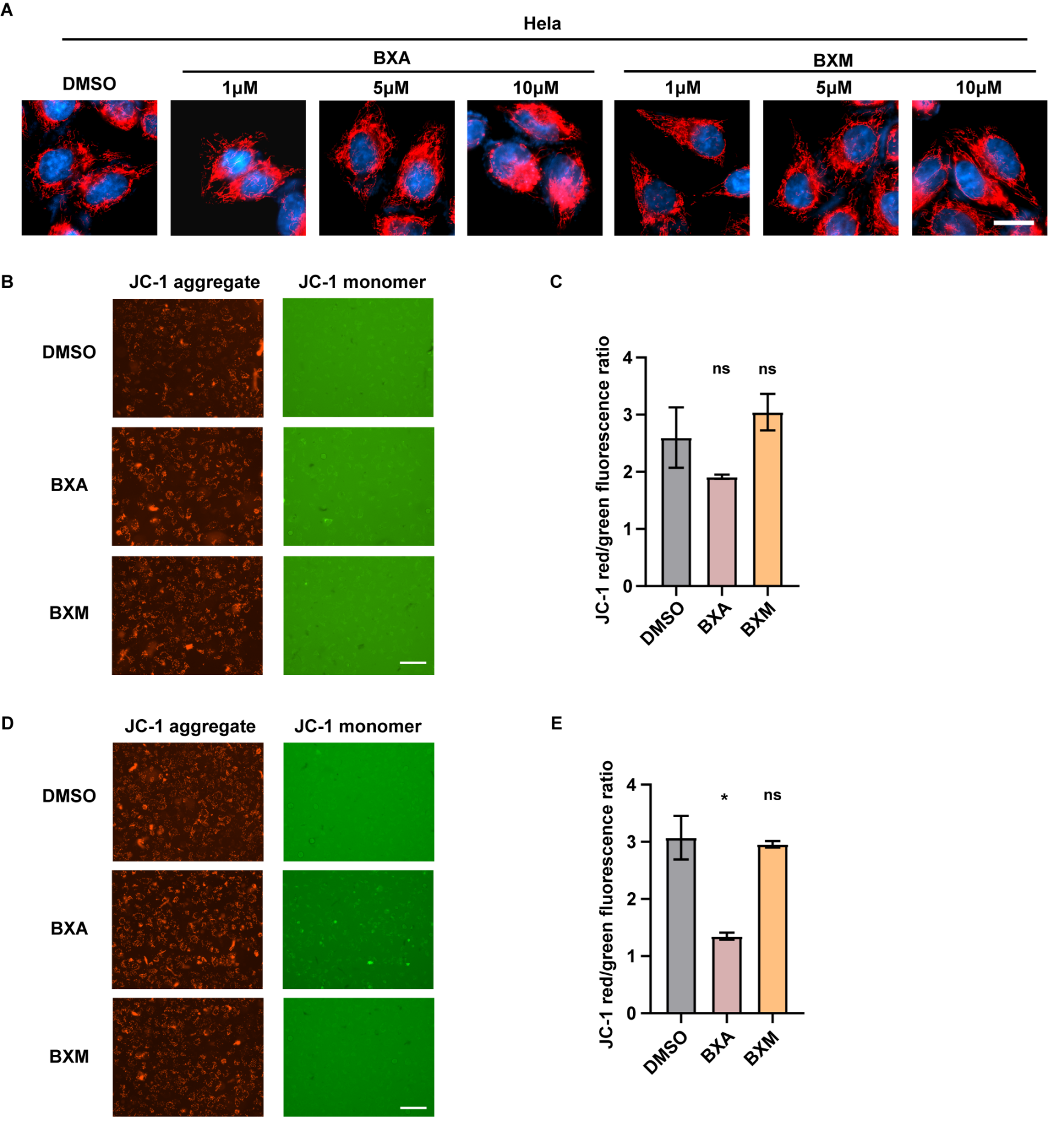

Supplement: Supplementary file 1 [file ijms-27-02967-s001.zip › Supplementary Figure 1.tif]

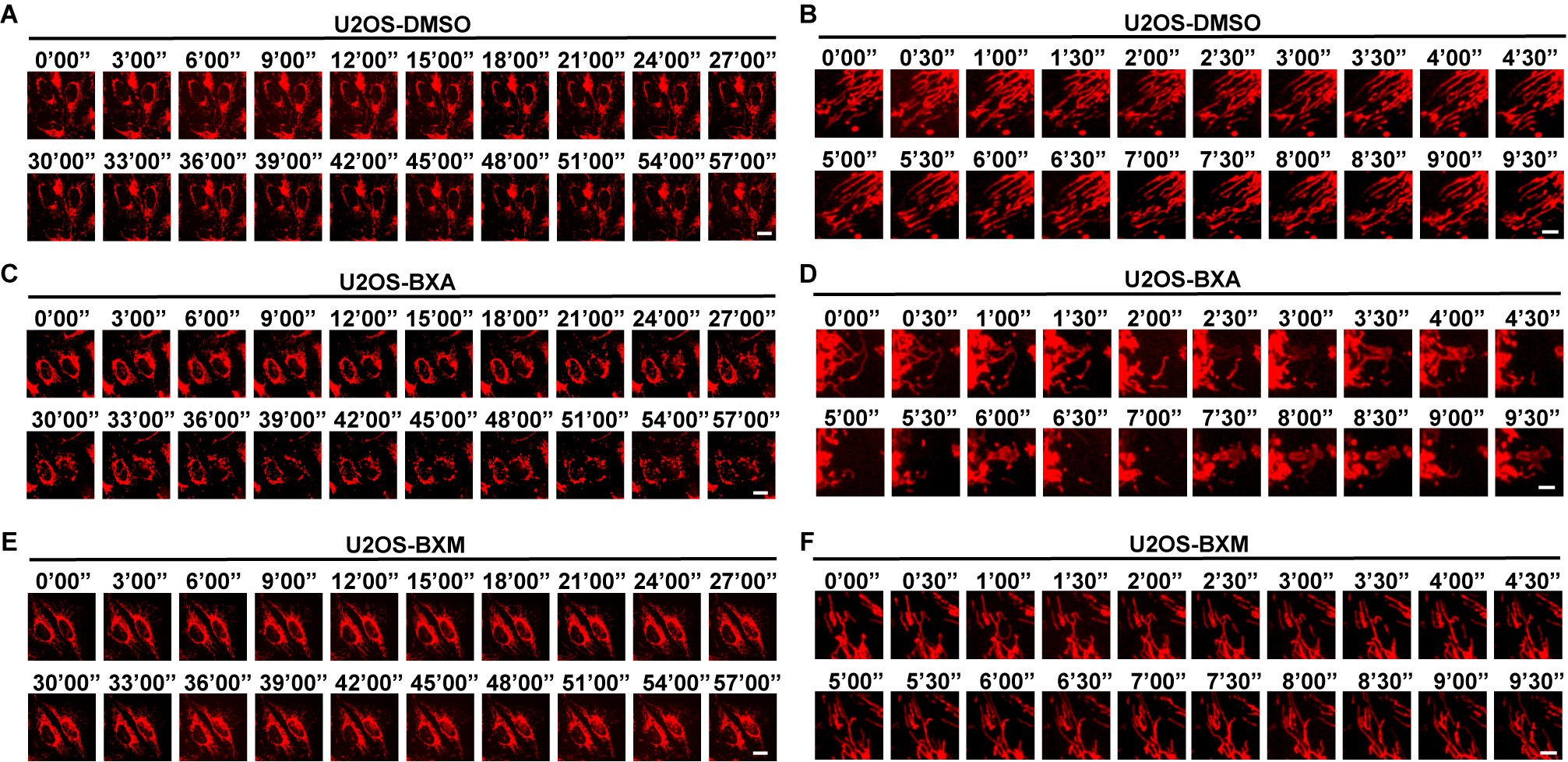

Supplement: Supplementary file 1 [file ijms-27-02967-s001.zip › Supplementary Figure 2.tif]

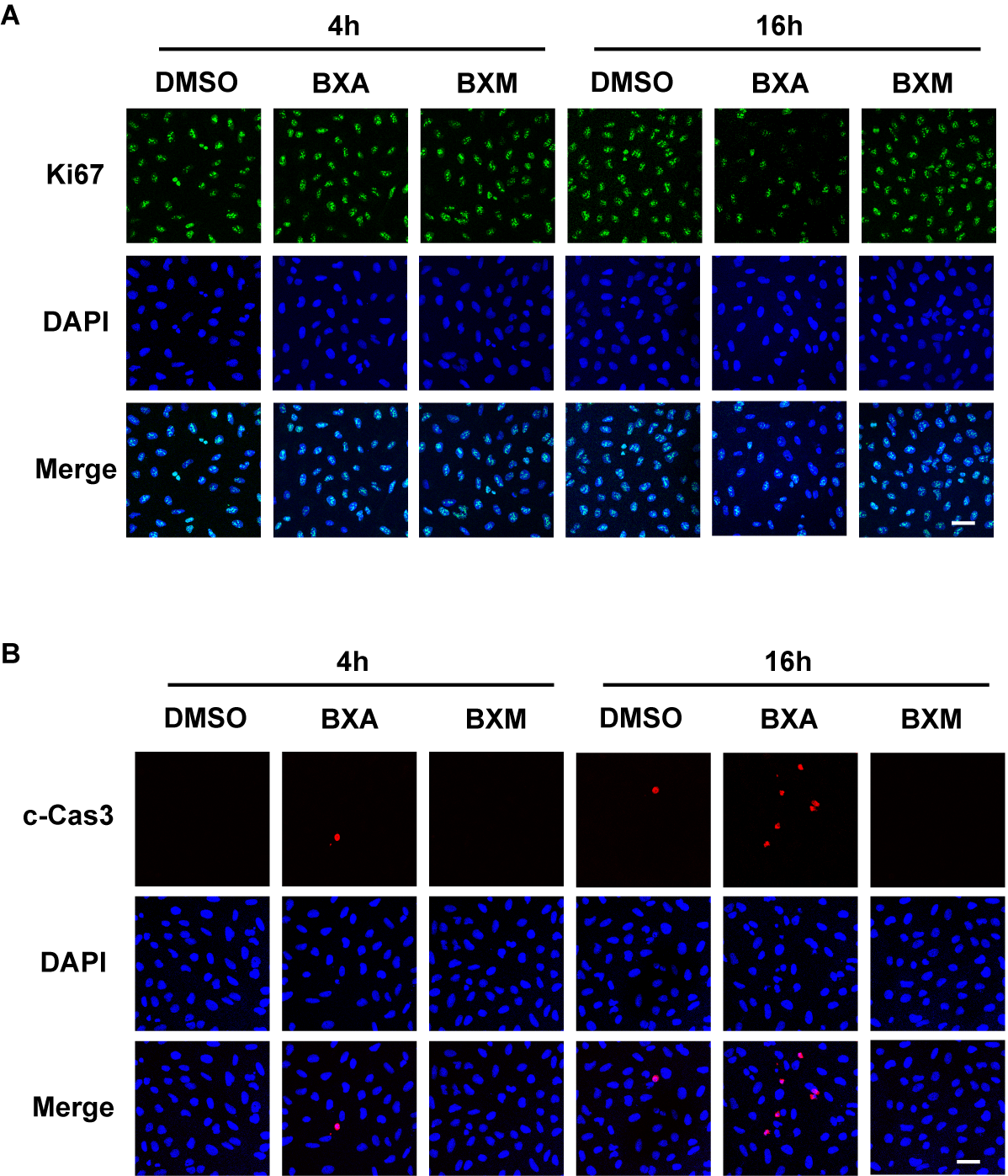

Supplement: Supplementary file 1 [file ijms-27-02967-s001.zip › Supplementary Figure 3.tif]

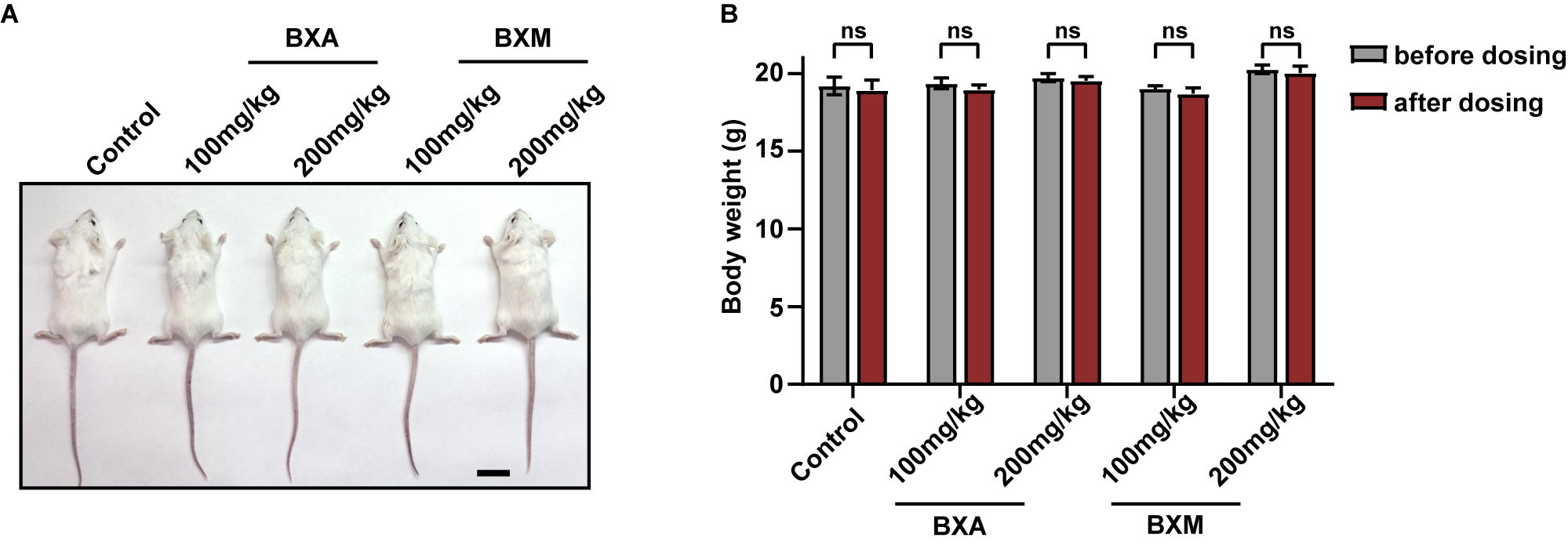

Supplement: Supplementary file 1 [file ijms-27-02967-s001.zip › Supplementary Figure 4.tif]
